# Development of Willow Tree Yield-Mapping Technology

**DOI:** 10.3390/s20092650

**Published:** 2020-05-06

**Authors:** Maxime Leclerc, Viacheslav Adamchuk, Jaesung Park, Xavier Lachapelle-T.

**Affiliations:** 1 Department of Bioresource Engineering, Faculty of Agricultural and Environmental Sciences, McGill University, Ste-Anne-de-Bellevue, QC H9X 3V9, Canada; maxime.leclerc3@mail.mcgill.ca (M.L.); jaesung.park@mail.mcgill.ca (J.P.); 2 Ramea Phytotechnologies, Saint-Roch-de-l’Achigan, QC J0K 3H0, Canada; xlachapelle@ramea.co

**Keywords:** precision agriculture, yield estimation, machine vision, willow tree

## Abstract

With today’s environmental challenges, developing sustainable energy sources is crucial. From this perspective, woody biomass has been, and continues to be, a significant research interest. The goal of this research was to develop new technology for mapping willow tree yield grown in a short-rotation forestry (SRF) system. The system gathered the physical characteristics of willow trees on-the-go, while the trees were being harvested. Features assessed include the number of trees harvested and their diameter. To complete this task, a machine-vision system featuring an RGB-D stereovision camera was built. The system tagged these data with the corresponding geographical coordinates using a Global Navigation Satellite System (GNSS) receiver. The proposed yield-mapping system showed promising detection results considering the complex background and variable light conditions encountered in the outdoors. Of the 40 randomly selected and manually observed trees in a row, 36 were successfully detected, yielding a 90% detection rate. The correctly detected tree rate of all trees within the scenes was actually 71.8% since the system tended to be sensitive to branches, thus, falsely detecting them as trees. Manual validation of the diameter estimation function showed a poor coefficient of determination and a root mean square error (RMSE) of 10.7 mm.

## 1. Introduction

Biomass has garnered much interest as an alternative sustainable energy source through combustion and transformation. Biomass comprises natural materials from living, or recently dead, plants, trees, or animals which are recycled as fuel in industrial production. More specifically, forest biomass consists of all parts of the tree, such as the trunk, bark, branches, needles, leaves, and roots [1]. At the basic level, photosynthesis allows trees to convert light energy, carbon dioxide, and water into biomass and oxygen. Since trees fix carbon while growing, the production of woody biomass demonstrates a neutral carbon balance when cultivated in low-quality areas such as marginal and abandoned fields. To maximize carbon fixation, research has focused on tree species that yield high amounts of rapid biomass production. Suitable species for biomass production include Willow (*Salix* spp.), Poplar (*Populus* spp.) and Eucalyptus (*Eucalyptus* spp.). Forest biomass can be processed as biofuel in a solid, liquid, and gaseous phase, which can then be burnt to generate useful energy, usually in the form of electrical energy [2].

As biomass production can positively impact sustainability in the energy industry, Precision Agriculture (PA) has had a positive impact on sustainability in the agricultural industry. In the case of crop production, this technology aims at implementing site-specific management (SSM) solutions for agricultural inputs [3]. By doing so, farmers can optimize their operations, reducing the effect of these inputs on the environment. PA technologies rely heavily on the Global Navigation Satellite System (GNSS), which can be used to locate agriculture vehicles in real-time in a field environment. One of the earliest and most common applications of GNSS-based systems is yield monitoring and mapping. Yield monitoring systems are designed to estimate crop yield and/or yield quality in real-time during harvesting [4]. The system is then able to link yield estimates to geographical coordinates to produce a yield map. The map is updated in real-time on a display that can be viewed by the operator. After harvesting, it can be retrieved for further analysis to ascertain its effectiveness in terms of performance, impact of management practices, locating low- and high-yielding zones, and so on. 

Although yield maps are essential tools for today’s agriculture, not all crops can be monitored for yield. Currently on the market, yield-monitoring systems are available for grain (e.g., corn, soybean, wheat), cotton, and sugarcane. Consequently, farmers growing other field crops (e.g., specialty crops, vegetables, fruits) must still rely on prior techniques to determine crop yield, where spatial resolution is far inferior to yield monitoring systems.

Methods have been proposed to estimate the yield of specialty crops based on optical feature detection [5,6,7,8]. The acquisition of image data using vision sensors, such as conventional cameras, can be analyzed in real-time or post-harvest by computer-vision (CV) algorithms to generate yield maps. The main advantage of optical feature detection is its flexibility since multiple types of feature can be extracted from an image such as color, texture, and shape. Yield-monitoring systems can be built with the capability of extracting distinctive features of a crop to use the output to produce a yield map.

A machine vision (MV)-based yield-monitoring system was developed for vegetable crops [6]. Vegetable segmentation was performed through the watershed algorithm and color data was used for classification. The system was designed to detect crop flow at the individual level, classify them according to shape and size, and count them. Testing was done with shallot onions, but the algorithm was constructed in a way to make it transferable to charlotte onions, Chinese radish, carrots, and lettuce. Results showed a coefficient of determination (R^2^) of 0.46 for the overall accuracy of the system. A single RGB camera was used and occlusion, as well as variable light conditions, were the limiting factors for this research.

An image segmentation framework for fruit detection and yield estimation in apple orchards had been investigated [7]. A spherical video camera capable of capturing a full 360° panoramic view was mounted on a remotely controlled testing platform. The framework included contextual information concerning relevant metadata to evaluate state-of-the-art convolutional neural network (CNN) and multiscale multilayered perceptions to perform pixel-wise fruit segmentation. Once the binary image was obtained, watershed segmentation followed by a circle Hough transform algorithm to detect and count the apples. Results showed that the combination of CNN pixel-wise classification and watershed segmentation produced a higher coefficient of determination (R^2^) at 0.83 compared to post-harvest fruit counts obtained from grading and counting machines.

An automated apple yield monitor using a stereo rig was demonstrated [8]. The system was mounted on an autonomous orchard vehicle fitted with a controlled artificial light source for nighttime data acquisition. Images from both sides of each tree row were captured. The algorithm started by hue and saturation segmentation, followed by the detection of local maxima in the grayscale intensity profile (i.e., specular reflection) as the feature indicating apples. Once detected, the intensity profile over four lines (i.e., going through the local maxima) of 21 pixels each was checked for roundness to remove false positives. Apples were then registered and counted as crop yield estimation. Errors of −3.2% and 1.2% in yield were reported for red and green apples, respectively.

A method for robust detection of trees using color images in a forest environment was published [9]. The proposed approach was characterised by a seed point generation algorithm followed by a multiple segmentation algorithm. Output segments were refined by morphological operations before being evaluated by a quality function based on the segment’s specific property (i.e., area of convex hull, area, orientation, eccentricity, solidity, and height). For each seed point, the segment with the best quality score was selected and all selected segments were fused in one frame which represented the final output of the tree detection algorithm. For that research, 30 images containing 197 trees were collected in a forest environment. The tree detection rate was reported as 86.8% with a corresponding precision of 81.4%.

The goal of this research was to develop a new yield mapping system for willow trees based on MV technology. The system was designed and built in partnership with Ramea Phytotechnologies, Inc. (Ramea), based in Saint-Roch-de-l’Achigan, Quebec. The system was designed to gather the physical characteristics of willow trees and tag them to their geographical coordinates on-the-go, while the trees are being harvested. Features assessed include the number of trees harvested and their diameter. Furthermore, the system has potential as a fast and non-destructive yield estimation tool which could be used to assess carbon dioxide (CO_2_) sequestration of willow trees grown in a short-rotation forestry (SRF) system over time.

Since methods for growing willows in SRF vary greatly depending on countries and regions [2], the developed willow tree yield mapping technology must be malleable enough to make this research relevant to industry partners and to producers around the world. Optical sensors have the capacity of sensing more than one feature such as color, intensity, geometry and texture [10]. Considering that the number of stems and morphological characteristics of the willow trees were assessed, and not bulk mass, the use of an optical, multi-sensing, platform was desirable. Moreover, for systems to be adopted in the agricultural industry, they must be affordable, rugged, and easy to install and manage. Thus, willow tree yield mapping based on MV was the preferred and selected method to accomplish the goals of this project.

## 2. System Components 

In terms of fruit detection and localization, several studies have been carried out to assess the performance of different vision sensor technologies. In doing so, researchers reported on the main issues involved in using camera sensors in an outdoor environment. Variations in light conditions, as well as variations of color, shape, and size of the target, are common problems that researchers face [10]. Moreover, complex plant canopy structures can create occlusion of targets, which makes detection even more challenging [11]. For this research, cameras using monochrome imagers were preferred to diminish the effect of variations in light conditions that are prominent in outdoor scenes. 

To improve target segmentation in the complex background environment, a camera featuring 3D vision was also favored to better extract the willow’s feature from the scenes. By using two imagers, it is possible to extract depth information from a pair of images. This technology is called stereovision. It aims to mimick the mammalian binocular vision system by collecting a stereo pair of images of a scene from two cameras with a known geometrical relationship. Using a matching algorithm, the system matches key-points of one stereo-pair image to the other. It then estimates the disparity between matching key-points and uses this to compute depth according to the system’s intrinsic and extrinsic parameters [12]. 

### 2.1. Sensors 

To acquire raw data, the RealSense D435 RGB-D camera from Intel (Intel Corporation, Santa Clara, California) and a Global Positioning System (GPS) receiver with Wide Area Augmentation System (WAAS) differential correction capability model 19x-HVS from Garmin (Garmin Ltd., Olathe, Kansas) were used. The RealSense D435 (Table 1) features infrared active stereovision technology to create depth maps using an embedded infrared projector. Its large Field Of View (FOV) makes it ideal for an outdoor setting with large targets at close range. The camera is fitted with one red green blue (RGB) imager and one stereo depth module containing two monochrome imagers. Images from the stereo depth module are processed by an embedded processor (Vision Processor D4) to generate depth maps. The RealSense can output four data streams at once in real-time: RGB stream, two monochrome imager streams, and the depth map stream. The on-board Vision Processor D4 makes this camera suitable for an embedded solution since it does not require the host computer to perform the heavy computation required by the stereo-matching algorithm.

For this research, a resolution of 1280 × 720 was used to obtain the best pixel-to-millimeter conversion factor possible. The infrared projector was turned off, since it is designed to improve performance in low texture scenes such as a dimly lit white wall [13,14].

### 2.2. Sensors Position and Mount

Early in the research, the front end of the tractor was considered to be the best location for the camera. The region of interest (ROI) for the willow trees was set between 3.05 m and 3.66 m measured from the soil surface. To achieve this with the RealSense FOV, the camera was mounted at 1.78 m from the ground surface and given a 28.5° tilt angle measured from the ground plane. The horizontal axis of the camera FOV was parallel to the row and the perpendicular distance between the camera and the row was 1.22 m. 

**Figure 1:**
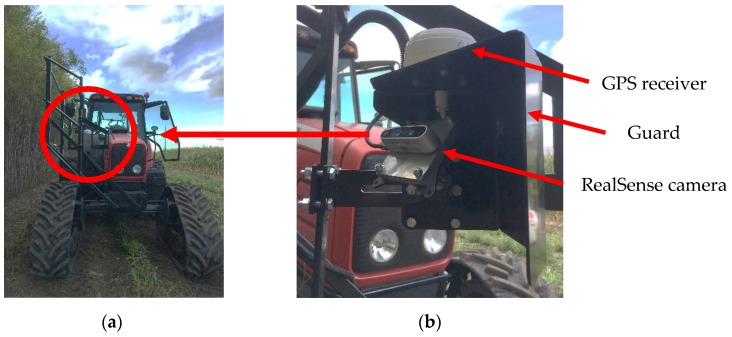
(**a**) Overall view of final camera position; (**b**) close view of final camera and Global Positioning System (GPS) mount.

The camera and GNSS mount (Figure 1) were designed as a multi-purpose assembly. In addition to providing mounting points for both sensors, it served as a guard against branches and sunlight. Branches can be expected to be leaning outside of the row if they have been hit while harvesting the adjacent row. Contact between a branch and the stereovision camera could result in loss of frame quality and physical damage to hardware (i.e., camera and data transfer cable). As the camera is tilted, direct sunlight can overexpose the optical sensors resulting in a significant decline of the frame’s information. The mount was designed to protect the camera when the sun is shining directly above. The sensor mount is also designed to allow easy fine-tuning of the camera position and angle within the field. Extra mounting points were also integrated to secure the assembly to the tractor in the event that future vibration became excessive. 

## 3. Willow Tree Algorithm

**Figure 2:**
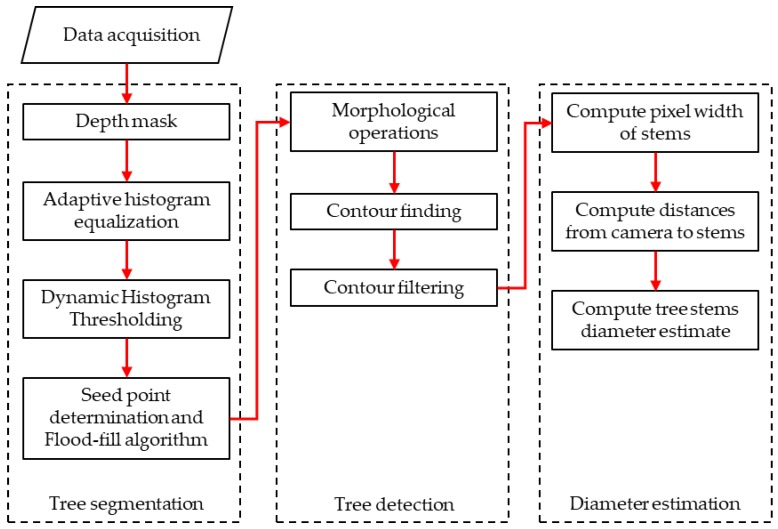
Willow tree algorithm flowchart.

The multiple steps that had to be taken in order to count the number of willow trees and to estimate their diameter for each frame are described below and summarized in Figure 2. From this point, when the term “monochrome imager” is used, it implies the leftmost imager of the RealSense D435 sensor (looking at the front view). Since the depth map is computed from this frame’s perspective of a scene, they are naturally aligned from one another and additional computation is avoided. The frame produced by the monochrome imager will be referred to as the “grayscale image”. 

Python was chosen as the programming language for the algorithm and was heavily dependent on NumPy and OpenCV libraries. As the RealSense camera is marketed to developers, a low-level software development kit (SDK) was freely provided by Intel through GitHub (GitHub, Inc., San Francisco, CA, USA) for the RealSense.

The Willow Tree algorithm runs using multiple parameters inputted in open-sourced and user-defined functions. It is relevant to mention that no formal procedures took place to optimize those values. For steps where parameters were involved, system performance and processing time were important criteria with special attention for overfitting since the system was not validated on an extensive data set. 

As this system is the first of its kind to the knowledge of the researchers (i.e., MV-based yield monitoring system for willow trees grown in SRF), it was of interest to see if classical CV operations and algorithms such as masks, histograms, adaptive histogram equalization and contours properties could achieve tree detection and diameter estimation with good results. More complex methods such as machine learning and deep learning were not examined for the development of this algorithm since potential problems with classical methods were not known for this application [15]. Furthermore, for the system to be adopted, the algorithm must be able to run on an embedded system, with finite computing power, and affordable for farmers while being designed for a harsh environment. 

### 3.1. Segmentation

To perform the segmentation, several steps were carried out using the depth map and the grayscale image to yield a segmented binary frame. The first was the computation of a depth mask, followed by the application of adaptive histogram equalization on the grayscale image. Then, both outputs were used as inputs in a dynamic histogram thresholding function where seed points were determined and input in the connected components algorithm flood-fill. The flood-fill algorithm output was the product of the segmentation procedure. 

#### 3.1.1. Depth Mask

The computation of a binary mask using the depth map was a necessary prior step to the dynamic histogram thresholding. The goal was to create a rough mask of the row being harvested to be input in the histogram calculation. This ensured that most of the background pixels were not considered in the histogram. A thresholding plane was first initialized to set the depth value for every pixel to zero if it was greater than the threshold plane at a specific location. Since the camera was tilted at 28.5° from the soil surface, the threshold plane was tilted by the same amount to account for a greater thresholding distance for the top pixel row compared to the bottom pixel row of the depth map. The top was computed as 2.33 m and the bottom was set as a constant at 1.23 m, which corresponds to the center of the row relative to the camera position. Using the vertical FOV of the monochrome imager, the height of a pixel in meters was computed based on the perpendicular distance from the camera to the threshold plane. Next, simple trigonometry was applied to find the length in meters of the hypotenuse formed between the camera center (fix), the bottom pixel row (fix) and the currently accessed row. This method was iteratively repeated until the top row was reached. This approach was found to be more computationally efficient than rotating the depth map itself.

Before inputting the depth map in the thresholding function, a median filter was applied to remove some of the noise with a 5 × 5 pixel square kernel. This type of filter works by replacing the kernel’s center value by the median of all pixels under itself [16]. It was effective at removing the within region noise while keeping the tree stems sharper than a standard Gaussian filter which was important to promote accurate diameter estimation. The equation below shows the final thresholding operation.

(1) $$\begin{array}{c} Depth\;mask{\;}_{i,j}=\{\begin{array}{c} 1\\ 0 \end{array}\;\begin{array}{c} {\mathrm{if}\;\mathrm{Depth}\;\mathrm{median}}_{i,j}<{\;\mathrm{Threshold}\;\mathrm{plane}}_{i,j}\\ \mathrm{otherwise} \end{array} \end{array}$$

#### 3.1.2. Adaptive Histogram Equalization

**Figure 3:**
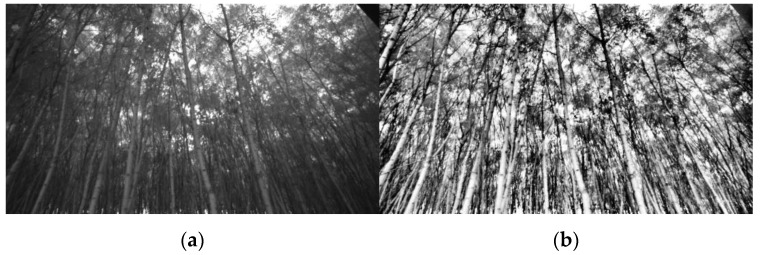
(**a**) Original grayscale image from the infrared imager; (**b**) grayscale image after applying the contrast-limited adaptive histogram equalization (CLAHE) algorithm.

Adaptive histogram equalization was used to increase the contrast in the grayscale image from the monochrome imager. The goal was to increase the intensity of the tree stem pixels relative to the background and non-tree foreground objects. The OpenCV implementation of the contrast-limited adaptive histogram equalization (CLAHE) algorithm was used. This algorithm was first introduced by Pizer, et al. [17] for post-processing of still medical images. It starts by dividing the input image into regions. There are three classes of regions: the corner regions (one in each corner), the border regions (adjacent to the image border), and the inner regions. Then, the CLAHE algorithm calculates the histogram of each individual region independently. From the function input parameters, a clip limit is calculated and applied to the histograms. Those histograms are then redistributed so as not to exceed the clip limit. Lastly, the cumulative distribution functions of the resultant contrast-limited histograms are determined and a linear combination of the four nearest regions are taken for grayscale mapping. The major drawback of this first implementation was its expensive computation. However, Reza [18] proposed another approach that made the CLAHE algorithm possible for real-time applications. CLAHE, with a clip limit of 40 and 8 × 8 pixel subregions, was applied as a pre-processing step before being input in the dynamic histogram thresholding function. The resulting frame is shown in Figure 3. CLAHE was used over non-adaptive histogram equalization methods as it yielded a frame with a greater contrast between tree objects and the background; thus, making trees easier to segment using the dynamic histogram thresholding methods explained in the next section.

#### 3.1.3. Dynamic Histogram Thresholding

A user-defined dynamic histogram thresholding function was implemented to find a list of pixel intensities that were most likely representing a willow tree in the CLAHE equalized frame. While developing the Willow Tree algorithm, it was noticed that when applying the depth mask to a histogram function of the corresponding grayscale image, the histogram region representing the tree pixel count spiked significantly compared to other regions. 

**Figure 4:**
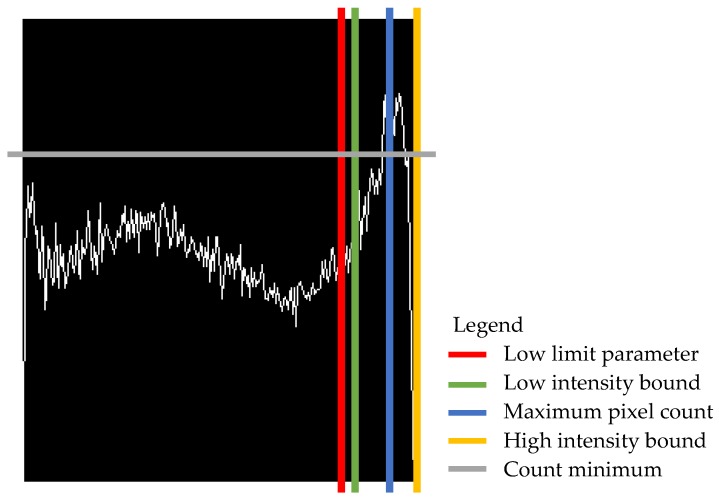
Histogram curve with thresholding parameters and boundaries displayed.

The function was designed to take five arguments (i.e., image, mask, peak range, low limit, count difference) and output a Python list of the intensity values (0 to 255 scale) that best represent the tree objects. The first step was to compute the histogram of the complete input image with one bin for every pixel intensity value and then to normalize the obtained histogram object from 0 to 255. Those two operations use functions already available in the OpenCV library. Next, the location of the maximum value was found, and the peak range parameter was applied where the central element was the maximum value of the normalized histogram object (Equation (2)). Figure 4 graphically represents the location of the parameters for the histogram of Figure 3b).

(2) $$\begin{array}{c} Intensity\;range\;boundaries=maximum\;pixel\;count\pm \frac{peak\;range\;}{2} \end{array}$$

In this case, the function was run with a peak range value of 50. A low and high clipping value equal to the low limit parameter and the total number of bins were applied to restrict the intensity range in one area of the histograms as well as handling run time errors in Python. The following step was to check if the normalized pixel count values found in the intensity range respect the pixel count difference relative to the maximum normalized pixel count found earlier. To perform this test, the difference in Equation (3) was computed. If the difference was greater than zero, the intensity value was added to the final list of pixel intensity values which best represent willow trees in the current frame.

(3) $$\begin{array}{c} Difference_{i}=intensity\;range_{i}-\left(maximum\;pixel\;count-count\;difference\right) \end{array}$$

(4) $$\begin{array}{c} Pixel\;intensity\;list_{i}=\{intensity\;range_{i}\;if\;difference_{i}>0 \end{array}$$

#### 3.1.4. Seed Point Determination and Flood-Fill Algorithm

In this final step of the segmentation procedure, the intensity values found in the previous step are used to find seed point coordinates to be used in the flood-fill algorithm. The output is a binary image where the white pixels represent the segmented areas that are most likely to be willow trees using depth and intensity information from the scene. The first step was to create a frame, called background removed, from which the seed points could be found. The following equation describes this operation.

(5) $$\begin{array}{c} Background{\;\mathrm{removed}}_{i,j}=\{\begin{array}{c} input\;image_{i,j}\\ 0 \end{array}\;\begin{array}{c} {\mathrm{if}\;\mathrm{Depth}\;\mathrm{mask}}_{i,j}\;\neq \;0\;\\ \mathrm{otherwise} \end{array} \end{array}$$

In this case, the input image is the same as with the previous step, which is the CLAHE equalized grayscale image. Next, for every intensity value in the pixel intensity list, the background removed image was scanned and the seed point image array is given a value of 1 at the location where a match is found.

(6) $$\begin{array}{c} Seed\;points_{i,j}=\{\begin{array}{c} 1\\ 0 \end{array}\;\begin{array}{c} {\mathrm{if}\;\mathrm{Backgroud}\;\mathrm{removed}}_{i,j}=Pixel\;intensity\;list_{k}\\ \mathrm{otherwise} \end{array} \end{array}$$

Using the argwhere() function in the NumPy library, a list of all (x,y) coordinates of the marked pixels in the seed point array was created. Finally, the coordinates were iteratively used in the OpenCV implementation of the flood-fill algorithm. Two other image arrays were given to the function; the CLAHE equalized infrared image and an empty mask. The grayscale image was used to compute the connected components of a seed point and for which they had their value set to 1 for the corresponding pixel in the mask. For two pixels to be connected, they must agree with the following statement:

(7) $$\begin{array}{c} Mask_{i,j}=\{\begin{array}{c} 1\\ 0 \end{array}\;\begin{array}{c} if\;input_{x,y}-lowdiff\leq input_{i,j}\leq \;input_{x,y}+updiff\\ otherwise \end{array} \end{array}$$

where, $input_{i,j}$ is the pixel intensity of the currently observed pixel and $input_{x,y}$ is the pixel intensity of the input image at the seed point coordinate. The parameters lowdiff and updiff are the maximal lower and higher brightness difference, respectively, between the currently observed pixel and its corresponding seed point. In this case, it is a fixed range implementation since the condition always refers to the original seed points [16]. For this research, a value of 10 and 30 for lowdiff and updiff, respectively, were used. 

**Figure 5:**
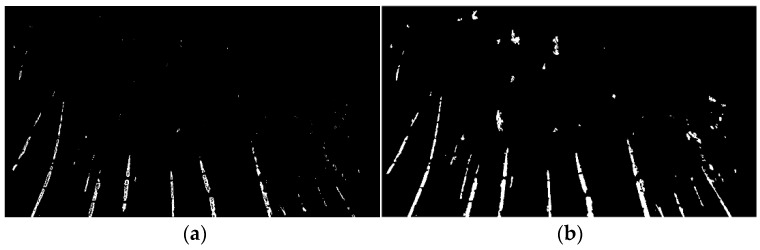
(**a**) Resultant seed point frame from the background removed image; (**b**) segmentation output binary frame.

More points were added to the mask as the function scan the list of seed point coordinates. The running time was limited by adding an if statement to check if a value of 1 was already assigned in the mask at the coordinate of the next seed point to be analyzed. This condition reduced the amount of time flood-fill would be run. For example, Figure 5a contains 11,300 points, but only 748 were used to produce the segmentation result in Figure 5b. After the iteration of all seed points, the mask array was reported as the final segmented binary image of the current scene

### 3.2. Tree Detection

In the detection procedure of the willow tree algorithm, a morphological closing operation was applied to the segmented binary image before using the OpenCV contour function to find all close contours that could represent willow trees. A user-defined function was designed to filter out unwanted contours and retain only tree contours under the same assumption. Contours that were still present after the filtering operation were considered by the algorithm as true detected willow trees. 

The morphological closing operation was undertaken to reduce noise that might have emerged in the flood-fill algorithm. It is commonly used after connected-components algorithms to remove elements resulting purely from noise and to connect nearby regions [16]. The closing operation is simply two morphological operations that are applied one after the other. The first is dilatation, where the pixel at the center of the kernel is replaced by the local maximum of all pixels covered by the kernel. For the binary image, the dilatation operation has the effect of “growing” each filled region. The second operation is erosion. It is simply the inverse of dilation, where the pixel at the center of the kernel is replaced by the local minimum of all pixels covered by the kernel. The effect of erosion on a binary image is to “reduce” the size of each region [16]. For this research, the kernel size used was a solid 2X15 kernel, applied with only one iteration. The kernel was set as an upward rectangle since it was the shape the target objects were expected to be. A morphological close was applied using the OpenCV implementation.

Once the noise had been removed, the binary image was input to the contour-finding function provided in the OpenCV library (i.e., *f*ind contours() function). It outputs a list of contour objects found in the binary frame. At the basic level, contour objects are a list of points that represent a curve within the image [16]. Contour object makes it possible to retrieve features about filled regions such as area, center, and bounding box. Those features were exploited to filter the raw contour list and find parameters for the true tree contours. 

**Figure 6:**
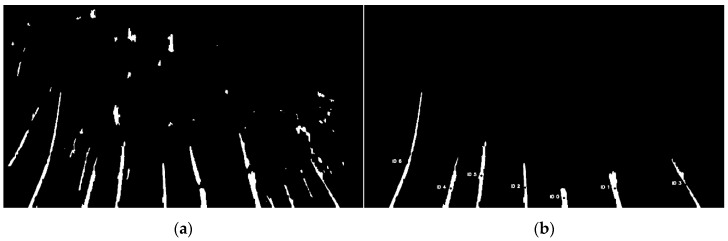
(**a**) Segmentation result after morphological closing was applied; (**b**) Detection procedure output frame.

Contour filtering used two criteria to remove non-tree contours. The first was the area to remove small filled regions that were most likely unwanted foreground objects or background residuals. The area of each contour was computed using a built-in function in OpenCV. A fixed threshold area of 600 was applied for this experiment. Next, it was assumed that if a contour is a tree, the contour must be present on the bottom pixel row of the frame, so it does not “appear” somewhere in the frame. To test this assumption, each contour was individually drawn in an empty image array. If all pixel values of the bottom row were equal to zero, the observed contour did not meet the requirement and was then removed from the contour list. Contours that were left in the list were considered as true detected willow tree regions. The willow tree algorithm then moves on to assign an identification (ID) number and to find the diameter of all detected trees within the frame. Figure 6b shows the final output.

### 3.3. Diameter Estimation 

Once the willow trees were detected, the algorithm proceeded by computing the diameter of the trees. As a first step, the pixel width of all remaining contours at the bottom row of the image array was found. Moreover, all trees were at a different distance from the camera, so the depth at this location was also found. Lastly, those features were fed into a function to find the pixel to millimeter ratio and the diameter in millimeters for all detected trees. 

To find the pixel width and depth of a detected tree, its corresponding contour was drawn in an empty image array. Then, the bottom pixel row of the drawn contour and the depth median frame were extracted. A new array, including both aligned rows, was created. The array was then trimmed on the column axis, where zeros were present in the contour row. The function traversed the row starting from both ends until it reached a non-zero element. From there, two possible cases were handled; in the first case, the contour row is solid (meaning no zeros within the trimmed array) and for the second case some zeros are present. 

Finding the pixel width and depth for the first case was straightforward. Once the zero columns were trimmed, the length of the array was saved as the pixel width and the depth was computed as the median of all depth values at the coordinates corresponding to the non-trimmed pixels. For the second case, the array was split in two at the location of the first zero encounters while traversing the contour row. Then, both sections were trimmed and traversed again for zeros. If no further zeros were found, the function recoded the pixel width and depth for every section. The section showing a greater pixel width was assumed to belong to the true tree. The consequence of handling all contours as in the first case would be to significantly overestimate the tree's diameter. 

As depth values did not always exist at the non-trimmed pixel coordinates, the zeros within the depth row were removed, so they did not affect the median result. In the particular case where all depth values were not available at the non-trimmed pixel coordinate, the median of the depth for all other contours in a specific frame was assigned. This was to be expected since depth values were not always available for all pixels due to low confidence within the RealSense stereo-matching algorithm. 

To compute the estimate of the tree's diameter, a function takes the argument found in the previous step (i.e., pixel width, depth) along with the depth scale and the FOV of the monochrome imager, which are a fixed constant acquired through the camera’s SDK. The depth scale was first used to convert the depth value of an observed tree from the camera scale to millimeters. Then, the half-width of the frame in millimeters for the converted distance was found. Using this value, the pixel-to-millimeter ratio was found for the observed tree. The final step was to multiply the pixel width with the obtained conversion factor. The steps described above to find any $tree_{i}$ diameter are shown by the following equation,

(8) $$\begin{array}{c} Distance_{i}\;\left(mm\right)=Depth\;value_{i}*Depth\;scale*1000 \end{array}$$

(9) $$\begin{array}{c} Half\;frame\;width_{i}\;\left(mm\right)=\tan\left(\frac{FOV_{x}}{2}\;*\;\frac{\pi }{180}\right)*Distance_{i} \end{array}$$

(10) $$\begin{array}{c} Ratio_{i}\;\left(\frac{mm}{pixel_{x}}\right)=\frac{Half\;frame\;width_{i}}{Frame\;size_{x}/2} \end{array}$$

(11) $$\begin{array}{c} Diameter_{i}\;\left(mm\right)=Pixel\;width_{i}*Ratio_{i} \end{array}$$

where *Depth scale* is the camera’s extrinsic parameter to convert the depth from the camera’s 3D coordinate system to meters; *Distance* is the distance between the tree and the camera in millimeters; $FOV_{x}$ is the camera’s FOV over the *x*-axis; *Half frame width* is the equivalent length of 640 pixels on the *x*-axis (i.e., half the normal resolution of 1280 pixels) in millimeters for a particular scene at $Distance_{i}$; $Frame\;size_{x}$ is the normal resolution on the *x*-axis in pixels (i.e., 1280 pixels); $Ratio_{i}$ is the pixel to millimeter conversion factor for $tree_{i}$ over the *x*-axis; and *Diameter* is the output diameter value for *tree_i_* in millimeters. 

### 3.4. Field Trials

All field trials were conducted in Ramea fields located in Saint-Roch-de-l’Achigan. Preliminary field trials were run in the first half of August 2019. The goal was to test the camera position as discussed and test for any hardware issues on site. In the final field trials, five classes of trees were defined, and eight trees were randomly selected per class. A total of 40 trees were observed from the same row on the same day. The classes were defined with the Ramea team. They represent their internal classification system for the manufacturing of green fences and noise barriers. Table 2 shows the morphological characteristics of all classes.

Two ranges for diameter were looked at: greater than 28.57 mm but smaller than 34.92 mm and strictly greater than 34.92 mm. Trees having a diameter less than 28.57 mm were not targeted for diameter characterization by Ramea, since they would be converted to mulch instead of being kept as rods for manufacturing green fences and noise barriers. It is essential to mention that diameter measurement for classification was taken at 152.4 mm from the ground surface. Diameter measurements for data validation were taken at 1.88 m from the soil surface, which was the real-world height of pixels located on the bottom row of the analyzed frame using a caliper with sub-millimeter accuracy. The stem diameter was measured on the plane parallel to the row line and perpendicular to the ground plane. Straightness was a subjective measure of how straight the stem was relative to itself for the 3.05 m to 3.66 m height section measured 152 mm from the ground surface. Table 2 describes where an excessive curvature is located relative to the height range of interest, if any. 

Each randomly chosen tree was identified using colored electrical tape as a marker. The electrical tape was applied at 1.5 m above the soil surface. A caliper was used to measure the stem diameter at 1.78 m and 0.15 m from the ground surface. As stems are not perfectly round in nature, the diameter was measured on a plane parallel to the tree row, which was coplanar to the plane used by the camera to estimate diameter. To ensure repeatability, a custom-built measuring stick was prepared to indicate the height where every measurement should be taken. This tool reduced the chance for errors as well as making data collection faster. The tool was used to adjust the tilt of the RealSense camera as well. Once it was placed straight up at the center of the row, the camera was tilted upward until the corresponding mark (3.81 m) on the stick could be seen in the monochrome imager output frame.

**Figure 7:**
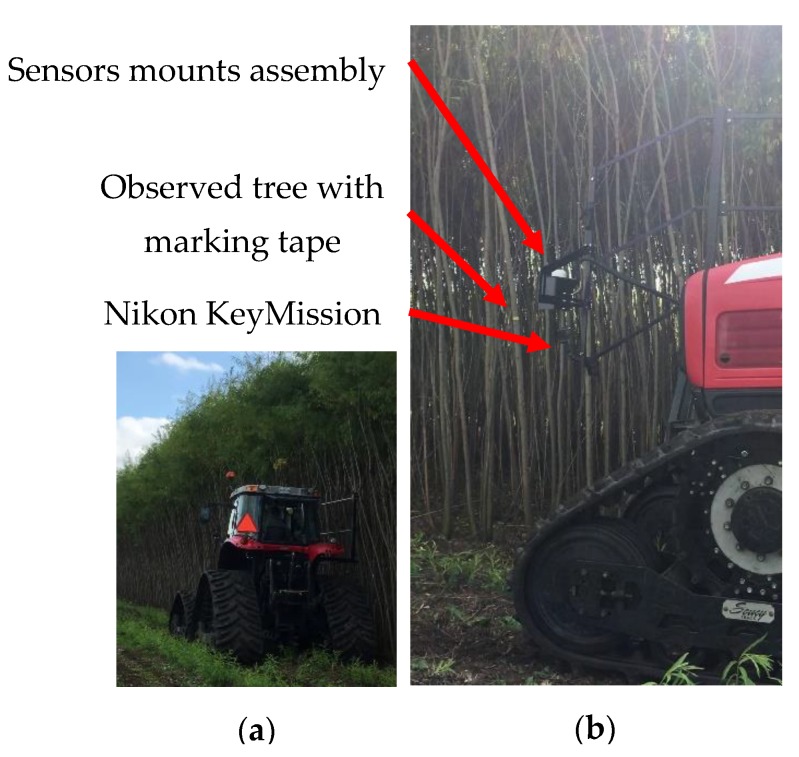
(**a**) Final field trials; (**b**) sensing components layout.

Data was then recorded by driving the tractor in front of the row as if harvesting were taking place. In order not to compromise the tree segmentation procedure, the colored electrical tape was placed below the RealSense camera FOV. To capture the markers, another camera, model KeyMission, featuring a wide FOV color sensor from Nikon (Nikon Corporation, Tokyo, Japan), was used. The latest model was able to capture the color markers in addition to the ROI of the RealSense. However, this technique required manual work to match the marked trees with the correct tree ID to perform data validation. As the Willow Tree algorithm was not ready for live testing during the harvesting season, all four streams of the RealSense were saved as one .bag file along with one text file containing raw GPS strings and one .mp4 file from the KeyMission for later analysis. Final field trials were conducted in late September 2019. The tractor was a Massey Ferguson (AGCO, Duluth, Georgia) model 6485 equipped with a track system form SoucyTrack (SoucyTrack, Quebec, Canada). The operator aligned the tractor center line with the center of the next parallel willow tree row allowing the camera to be at roughly 1.22 m from the center of the row as designed. The data acquisition was carried out at a speed of 2.3 km/h with the engine crank-shaft speed set at 2200 rotation per minutes (RPM). Those parameters are typical for the harvesting operation at Ramea. Figure 7 shows the complete hardware installed during the final field trials carried out on a sunny afternoon with little cloud cover.

### 3.5. Data Analysis

To assess the performance of the tree-detection procedure, 10 labeled images out of the 37 images containing the 40 observed trees were randomly selected and manually validated. Additionally, stratified sampling of frames with respect to the number of detected trees was undertaken. Two categories were created, frames containing 7 or less detected trees and frames containing 10 or more detected trees. For each category, 3 frames were randomly selected, so a total of 6 frames were selected as stratified samples. Thus, the complete set of images for which manual validation has been performed was 16 out of 37 images from the same pass in the field (i.e., 10 random samples and 6 stratified samples). This was done to better capture the distribution of the number of detected trees across frames for statistical analysis. Three variables were examined in each frame: number of correctly detected trees, number of falsely detected trees, and number of undetected trees. Since it can be ambiguous to distinguish trees and branches visually, clear criteria had been specified to count a tree as a tree and not a branch (and vice-versa). Criteria are defined in Table 3. 

The performance of the diameter estimation procedure was assessed using the 40 observed trees in Ramea fields. They corresponded to eight randomly selected trees per class for the five classes observed in the same row and on the same day. Since two ranges of diameters are examined within all classes (i.e., 28.57 mm < d < 34.92 mm, 34.92 mm < d), classes with the same range were grouped together. Thus, results from Class 1 and Class 2 are shown together as well as Class 3, Class 4, and Class 5. Root mean square error (RMSE) was computed according to class group as well as for all data points together. A linear regression with intercept fix at the origin between the observed and estimated diameter was undertaken without regard to class. Moreover, the two highest residuals (i.e., above and below regression lines) were removed from the dataset for further analysis. Hence, they were not included in the linear regression. 

## 4. Results and Discussion

### 4.1. Hardware Reliability

During the field trials, no issues related to hardware occurred. Mechanical and electrical connections were sturdy, and the sensor mount did not show excessive vibration. No loss of power, connection fault with sensors or loss of data took place. Considering the relatively high machine vibration due to the installed track system, the system hardware proved to be rugged and reliable.

### 4.2. Tree Detection Results

**Figure 8:**
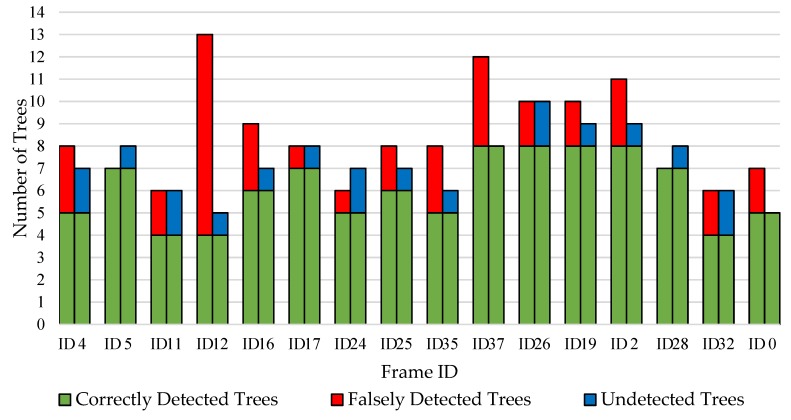
Manual validation compared to algorithm results.

Manual validation results of the tree detection procedure are shown in Figure 8. From the 16 images, a total of 135 trees were detected where 71.8% were correctly detected and 28.2% were falsely detected. Furthermore, 19 trees were not detected, which represents 16.4% of the total detected count. However, from the falsely detected group, 89.7% were branch objects and the remaining were clusters of trees for which the algorithm filled in the same contour. To support those results, it was also found that 90% of the 40 observed trees were correctly detected. This means that the algorithm was more sensitive than expected to branch objects. From the manually validated images, no non-tree or non-branch objects had been detected, which indicated that the segmentation procedure was able to properly remove the background and none-tree like objects in the foreground. On the horizontal axis of Figure 8, ID 4 to ID 37 are the 10 randomly sampled frames out the original 40 frames dataset. To the right of ID 37, ID 26 to ID 0 represents the 6 stratified sampled frames.

Undetected trees were mostly due to fragmented filled regions, which in fact, represented a single tree. Since the contour filtering function deletes regions smaller than a certain threshold before confirming its presence on the bottom pixel row, if only a small region was observed, the entire contour was deleted. Thus, all other contours representing the same tree were deleted as well and the tree was not detected. The Willow Tree algorithm has no features to regroup fragmented contours of the same object.

Looking at the distribution of the undetected trees within the sample of 40 observed trees, one tree per class was undetected, excluding Class 5, which equates to a 90% detection rate. Table 4 summarizes those findings.

**Figure 9:**
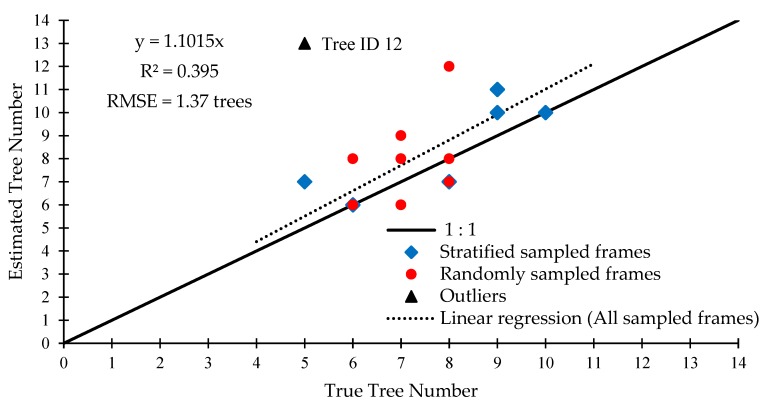
True tree count vs. estimated tree count per frame.

To examine detection precision, a linear regression between the true number of trees and the estimated number of trees was undertaken with the same 10 randomly sampled and the 6 stratified sampled frames (Figure 9). As can be seen, the algorithm tends to overestimate the count of true trees in frames. Knowing that the system has a high amount of falsely detected trees due to branches, this behavior was to be expected. ID 12 had the highest residual from the fitted regression line and so, it was not included in the statistical analysis but still shown as an outlier in Figure 9. The resultant linear regression features an R^2^ of 0.40 and a RMSE of 1.37 trees.

High falsely detected tree rates were mainly due to branches being detected as trees. The willow tree algorithm was sensitive to branches that were the closest to the camera since they could be picked up by depth maps; hence, their pixel intensity value was included in the determination of seed point coordinates to be input in the flood-fill algorithm. In the detection procedure, some of the filled regions representing branches were not filtered out since they were large enough and present on the bottom of the pixel row. Thus, improvement in contour filtering is needed to handle such cases without diminishing the number of correctly detected trees. One solution would be the implementation of a machine-learning technique to improve tree classification. Contour features could be fed into a classifier, such as support vector machine (SVM), which would be able to distinguish between contours representing trees and branches. SVM classifier has demonstrated a high accuracy rate compared to other machine-learning algorithms for fruit detection in outdoor scenes [10]. However, to create a robust machine-learning model, training data would need to be acquired at a different time of the day (e.g., morning, afternoon), in different weather conditions (e.g., sunny, cloudy), and at a different time of the year (i.e., spring, summer, fall). This, of courses, requires more time and resources but have the potential to improve results.

### 4.3. Diameter Estimation Results

**Figure 10:**
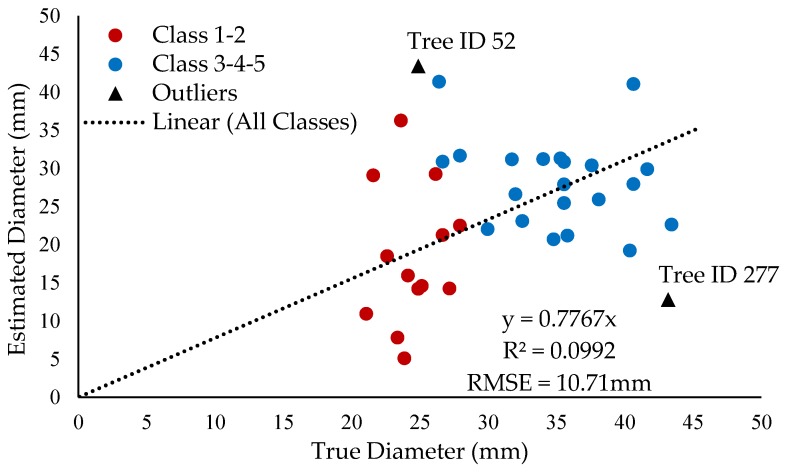
Measured tree diameter compared to estimated tree diameter.

Due to detection performance, it was possible to analyze 36 of 40 data points. The slope of the regression was found to be 0.78, which indicated that the function tends to underestimate tree diameter. The RMSE for the full dataset was found to be 10.7 mm, which represents 37.5% and 30.7% of the low and high diameter range boundaries, respectively. RMSE for group Classes 1–2 and Classes 3–4–5 was 10.6 mm and 10.8 mm, respectively. The overall coefficient of determination R^2^ was found to be poor at 0.099 as shown in Figure 10. 

**Figure 11:**
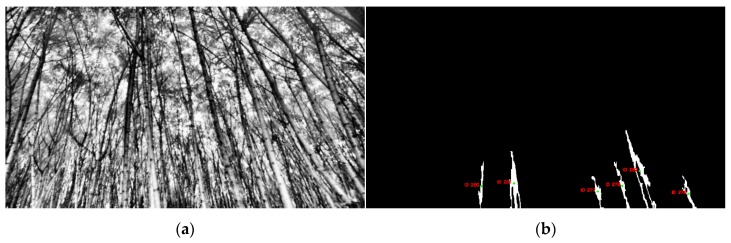
(**a**) CLAHE equalized frame; (**b**) segmentation procedure output with labeled detected trees.

Tree ID 277 and Tree ID 52 were the furthest outliers from the fitted regression line for the lowest and highest diameter estimation, respectively. Analyzing the situation at these points, provided insight into the system behavior. For Tree ID 277, the system output was 12.8 mm in diameter, where the measured diameter was 43.2 mm. After the diagnosis, a possible cause was found to be the poor quality of CLAHE equalized grayscale images. CLAHE equalized frames are used as input in the dynamic histograms thresholding function to compute the histograms and find seed point coordinates. In this case, some parts of the trees within the CLAHE frame were darker. Since the flood parameters in the flood-fill algorithm are taken as constant, the function was not able to connect the darker components to the final segmented binary image. Other contours of the same scene appeared skeletal as well. Figure 11 displays the CLAHE frame and the filtered contours frame. 

For Tree ID 52, the system output a diameter of 43.4 mm whereas the measured diameter was only 24.9 mm. Again, a diagnostic of the processed frames was conducted. In contrast to the previous case, here, the CLAHE equalized was too bright in the background, leading to the non-tree object being connected to a pixel pertaining to true tree objects as the clear distinction in pixel intensity was more subtle. This effect caused the filled region to be larger and hence, overestimated the diameter. 

Even if the use of color images was avoided to increase the robustness of the algorithm in varying light conditions, the effect was not completely prevented with grayscale images. Since the RealSense was tilted upward to improve depth map quality, it was also prone to over saturation when beams of light found their way through the tree canopy and into the camera’s optical sensors. To address this problem, the low and high difference parameters used by the flood-fill algorithm could adapt dynamically to current frames. It is possible that intensity location of the peak count value in the dynamic histogram thresholding function could be used as an indicator. 

### 4.4. Row Transect

**Figure 12:**
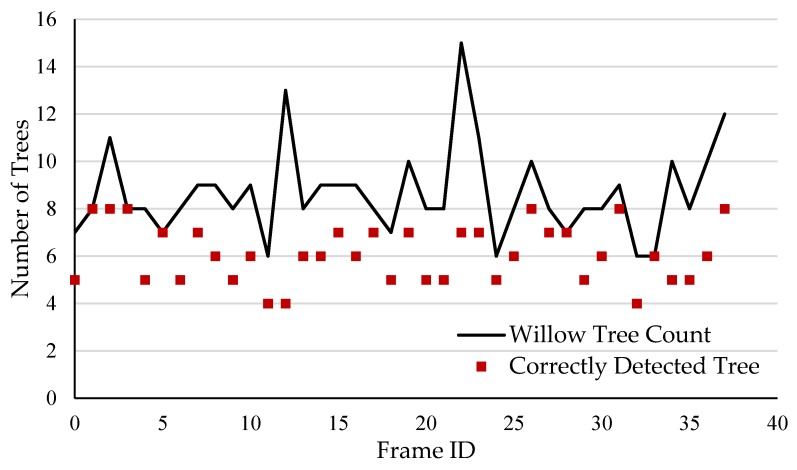
Willow tree yield transect.

Because data were acquired for a single willow tree row, spatial interpolation of willow tree count would not have been appropriate. Instead, a transect graph (Figure 12) was produced to show the variability of yield (i.e., number of trees) along the row. Since the operator must stop harvesting to unload before moving on to the next load, data collection must stop once for every cycle. To produce a yield map for an entire field, all data from single loads need to be merged and analyzed together. A spatial interpolation using the inverse distance weighting method could then be used.

## 5. Conclusions

A willow tree yield-mapping technology was developed based on the number and diameter of tree stems harvested. An RGB-D camera using stereovision technology was implemented to collect grayscale images and compute the depth maps of willow tree row scenes grown in a SRF system, prior to harvesting. The system also has potential as a fast and non-destructive yield estimation tool which could be used to assess carbon dioxide (CO_2_) sequestration of willow trees grown in SRF over time. Data from a GNSS receiver were integrated to tag image data to their corresponding geographical coordinates. A CV algorithm was developed to detect willow trees in images and estimate their diameter. To achieve this, the algorithm relied on equalized grayscale images using the CLAHE algorithm and depth maps information. The RGB stream of the camera was not used. The system was designed and built in partnership with Ramea Phytotechnologies, Inc.

The system was able to correctly detect 71.8% of tree stems and estimate their diameter with an RMSE of 10.7 mm. To achieve this, the willow tree algorithm relied on equalized grayscale images using the CLAHE algorithm and depth map information. Detection errors were primarily due to the detection of branches as tree objects which represented 89.7% of the number of falsely detected trees. Diameter estimation errors were mostly due to the low contrast between pixels pertaining to tree objects and backgrounds despite the implementation of the CLAHE algorithm. To increase system performance, the contour filtering procedure needs to be strengthened and the system must be able to better handle cases of oversaturation in scenes. This system is the first of its kind and provides a promising first step for further development of a more robust and commercially viable product.

## Figures and Tables

**Table 1:** RealSense D435 camera specifications.

Depth Technology	Active IR Stereo
Depth Stream FOV (Horizontal × Vertical × Diagonal)	87° ± 3° × 58° ± 1° × 95° ± 3°
Depth Stream Resolution	Up to 1280 × 720
Depth Stream Frame Rate	Up to 90 fps
Minimum Depth Distance (Min-z)	0.1 m
Maximum Depth Range	Approximately * 10 m
RGB Sensor FOV (Horizontal × Vertical × Diagonal)	69.4° × 42.5° × 77° ± 3°
Connector	USB-C 3.1 Gen 1

* Varies depending on calibration, scene, and lighting condition.

**Table 2:** Willow tree classification classes.

Class Number	Diameter	Straightness	Marker
1	28.57 mm < d < 34.92 mm	Curvature < 3.05 m	White
2	28.57 mm < d < 34.92 mm	Curvature > 3.05 m	Green
3	34.92 mm < d	Curvature < 3.05 m	Blue
4	34.92 mm < d	3.05 m < Curvature < 3.66 m	Yellow
5	34.92 mm < d	Curvature > 3.66 m	Red

**Table 3:** Tree detection validation criteria.

Criteria Number	Criteria
1	Trees must be visible in the grayscale’s bottom pixel row as one complete solid stem.
2	The stem must not be clustered in the same contour to be considered as correctly detected trees.
3	Stems must be at least 10 pixels wide at the haft height of the frame.

**Table 4:** Results of tree detection of the 40 observed trees.

Number of Trees	Tree Class				
	1	2	3	4	5
Observed	8	8	8	8	8
Correctly detected	7	7	7	7	8
Undetected	1	1	1	1	0
